# A New Chimeric Antibody against the HIV-1 Fusion Inhibitory Peptide MT-C34 with a High Affinity and Fc-Mediated Cellular Cytotoxicity

**DOI:** 10.3390/biology13090675

**Published:** 2024-08-29

**Authors:** Svetlana V. Kalinichenko, Lama Ramadan, Natalia A. Kruglova, Konstantin I. Balagurov, Marina I. Lukashina, Dmitriy V. Mazurov, Mikhail V. Shepelev

**Affiliations:** 1 Center for Precision Genome Editing and Genetic Technologies for Biomedicine, Institute of Gene Biology Russian Academy of Sciences, 119334 Moscow, Russia; natalya.a.kruglova@yandex.ru (N.A.K.); kostya.chamomilla@gmail.com (K.I.B.); dmitrii.mazurov@yale.edu (D.V.M.); 2 Institute of Gene Biology Russian Academy of Sciences, 119334 Moscow, Russia; lamaadnanramadan@gmail.com; 3 Dmitry Rogachev National Medical Research Center of Pediatric Hematology, Oncology and Immunology, 117997 Moscow, Russia; mlukashina@mail.ru

**Keywords:** chimeric antibody, HIV-1 fusion inhibitors, MT-C34 peptide, antibody-dependent cell-mediated cytotoxicity

## Abstract

**Simple Summary:**

HIV-1 is a hard-to-eradicate persisting infection which, despite the current antiretroviral therapy, takes about 600,000 human lives every year. HIV-1 uses the CD4 receptor and co-receptors CCR5 or CXCR4 on the surfaces of T cells, macrophages, and dendritic cells to enter the host immune cells. Inhibition of HIV-1 entry is one of the most effective approaches for blocking viral infection. Creating genetically engineered cells that are insensitive to HIV-1 entry is a promising instrument for virus eradication. We previously showed that knock-in-based expression of the fusion inhibitory peptides MT-C34 or 2P23 fully protected primary CD4 human T lymphocytes from HIV-1 infection. Here, we generated and characterized a novel human chimeric antibody against the MT-C34 peptide with the ability to mediate antibody-dependent cell cytotoxicity (ADCC). The created antibody is a useful supplementary reagent for the detection and enrichment of engineered HIV-1-resistant cells developed for research studies or clinical applications. Its ADCC activity can potentially be used for subsequent elimination of engineered malignancy-prone T and CAR cells in vivo, but the efficacy and limitations of in vivo antibody application should be determined further.

**Abstract:**

Peptides from heptad repeat (HR1 and HR2) regions of gp41 are effective inhibitors of HIV-1 entry that block the fusion of viral and cellular membranes, but the generation of antibodies highly specific for these peptides is challenging. We have previously described a mouse hybridoma that recognizes MT-C34-related peptides derived from HR2. It was used for the selection of HIV-1-resistant CD4 lymphocytes engineered to express the MT-C34 peptide via a CRISPR/Cas9-mediated knock-in into the *CXCR4* locus. In this study, we cloned variable domains of this antibody and generated a recombinant chimeric antibody (chAb) by combining it with the constant regions of the humanized antibody Trastuzumab. The new chAb displayed a high specificity and two-fold higher level of affinity than the parental mouse monoclonal antibody. In addition, chAb mediated up to 27–43% of the antibody-dependent cellular cytotoxicity towards cells expressing MT-C34 on their surface. The anti-MT-C34 chAb can be easily generated using plasmids available for the research community and can serve as a valuable tool for the detection, purification, and even subsequent elimination of HIV-1-resistant CD4 cells or CAR cells engineered to fight HIV-1 infection.

## 1. Introduction

HIV-1 enters cells using the CD4 receptor [1] and one of the co-receptors CCR5 [2,3] or CXCR4 [4], which are expressed on host immune cells such as T cells, macrophages, dendritic cells [5], as well as on CD34^+^ hematopoietic stem and progenitor cells (HSPCs) [6,7,8]. The replication of HIV-1 induces apoptosis [9] and pyroptosis of CD4^+^ T cells [10], and, if untreated, eventually leads to the development of acquired immunodeficiency syndrome (AIDS), opportunistic infections, and cancers [11]. Latently infected long-living memory CD4^+^ T cells, as well as CD34^+^ HSPCs, represent a main reservoir of HIV-1 in organisms, which is difficult to eradicate [8]. Despite the development of precise “DNA scissor” technologies, the excision of proviral DNA or knocking out of CCR5 co-receptors did not result in a complete eradication or functional cure of HIV-1 in clinical trials, largely due to insufficient efficacy, problems with molecular instrument delivery, or viral co-receptor switch [12].

Inhibition of entry is one of the most efficient ways to fight viral infection. The viral surface protein of enveloped viruses is usually trimer that, upon receptor/coreceptor engagement, triggers a release of fusion peptide from transmembrane protein and its insertion into the target membrane. However, to complete viral and cellular membrane fusion, the N-terminal heptad repeat (NHR) or HR1 domain should interact with the C-terminal heptad repeat (CHR) or HR2 domain and form a six-helix bundle, which brings two membranes in close apposition. Peptides derived from HR domains of the gp41 glycoprotein, especially from HR2, are potent inhibitors of HIV-1 fusion whose activity does not depend on coreceptor usage [13]. Two fusion inhibitory peptides, T-20 (Enfuvirtide or Fuzeon) and C34 (Sifuvirtide) were approved for clinical usage in the US and China, respectively, and only in combination with highly active antiretroviral therapy (HAART) [14,15], as a monotherapy with these peptides is not enough to control viral infection. Unlike soluble peptides, membrane-bound peptides demonstrated several orders of magnitude higher inhibitory activity [16,17,18]. This encouraged researchers to develop an alternative approach for gene-based delivery of glycosylphosphatidylinositol (GPI)-anchored peptides. Some of these GPI-anchored peptides, such as a long C46, were tested in clinical trials alone [19] or in combination with a short hairpin RNA (shRNA) against CCR5 (ClinicalTrials.gov ID NCT01734850) [20,21], or C34 peptide N-terminally fused to CXCR4 entered phase I (ClinicalTrials.gov ID NCT03020524) [21]. These studies demonstrated that peptides delivered to T cells or HSPCs using lentivirus had moderate effects on the CD4 count and viral load. However, the effectiveness of therapy could be improved if the immunoselection of modified cells is included in the protocol.

We have recently developed a CRISPR/Cas9-based fusion inhibitory peptide knock-in (KI) into the *CXCR4* locus and showed that the MT-C34 peptide protects the CEM/CCR5 T cell line or primary CD4^+^ T lymphocytes from HIV-1 infection when expressed on their surface in a context of a GPI-anchored protein CD52 [22]. MT-C34 has two additional amino acid residues, Met and Thr, at the N-terminus of the C34 peptide, which increases its binding to the HR1 domain of the gp41 protein and stabilizes the resulting complex [23]. We also explored the effect of HR2-derived and modified peptide 2P23 [24] alone or in combination with MT-C34 and showed that preselection of gene-edited lymphocytes with anti-peptide antibodies is required to demonstrate complete resistance of MT-C34^+^ and 2P23^+^ CD4 cells and their selective expansion during HIV-1 replication.

Antibodies are a useful tool not only for ex vivo cell selection but also for in vivo depletion of genetically modified lymphocytes with high malignancy potential, for example, CAR cells. To achieve elimination, either inducible suicide genes [25,26,27,28] or an addition of a selective marker to the CAR construct with an infusion of a cognate human antibody [29,30,31,32] proved their efficacy in clinics. Applying one fusion peptide recognized by one antibody for both cell protection, selection, and elimination is a new and attractive idea to keep HIV-1 gene therapy more efficient and less dangerous.

In this study, we aimed to improve an antibody against the MT-C34 peptide through the generation of a chimeric antibody (chAb) between the variable domains of the C24-6 mouse hybridoma that we obtained previously [22] and the human Fc-fragment of Trastuzumab (Tz) IgG1. This modification should enable the effector function of the anti-MT-C34 antibody in the human system and reduce its immunogenicity. The results of the cloning procedure and fusion with the human Ab showed that the new chAb retained specificity and acquired a higher affinity to the MT-C34 peptide in comparison to the mouse antibody. As a result of chimerization with the human Fc-fragment, chAb was able to mediate antibody-dependent cellular cytotoxicity (ADCC) when used with human peripheral blood mononuclear cells (PBMCs). This feature will expand the range of the application from just being a selection reagent to an instrument for the killing of peptide-modified cells, which we discuss in this paper. As we made it available, the new recombinant chAb will be not only an important asset in our CRISPR/Cas9-based peptide knock-in technology but also a helpful reagent for other researchers who develop gene-editing and CAR-based therapeutics to combat HIV-1.

## 2. Materials and Methods

### 2.1. Cell Culture

HEK293T cells were grown in DMEM medium (PanEco, Moscow, Russia) supplemented with 10% fetal bovine serum (FBS, #FB-1001/500, Biosera, Cholet, France), 2.5 mM of glutamine (PanEco, Moscow, Russia), and 10 µg/mL of gentamicin (PanEco, Moscow, Russia). CEM/CCR5 and CEM/CCR5/MT-C34 cell lines (further designated as CEM/R5 and CEM/R5/MT-C34, respectively) were generated by stable lentiviral transduction of the CCRF-CEM cell line (Cellosaurus CVCL_0207, purchased from the ATCC, Manassas, VA, USA), as described in [22], and were grown in RPMI 1640 medium (RPMI-XA, Capricorn scientific, Ebsdorfergrund, Germany) supplemented with 10% FBS, 2.5 mM of glutamine, and 10 µg/mL of gentamicin. C24-6 hybridoma cells [22] were grown in HybriS-2 medium (PanEco, Moscow, Russia).

### 2.2. Isolation of PBMCs

Human PBMCs were isolated from the peripheral blood of healthy donors by centrifugation on a Ficoll gradient (PanEco, Moscow, Russia) using a standard technique. To cryopreserve PBMCs (5 × 10^7^ cells/mL), they were resuspended in FBS containing 5% dimethyl sulfoxide and stored in liquid nitrogen until use. Before usage, cryopreserved PBMCs were quickly thawed at 37 °C and washed twice with 20 mL of RPMI growth medium.

Experiments that involved human blood samples were conducted in accordance with the Declaration of Helsinki and approved by the Human and Animal Ethics Committees of the Institute of Gene Biology Russian Academy of Sciences (Moscow) (protocol № 3 from 15 November 2023). Informed consent was obtained from blood donors for the use of their samples for research purposes.

### 2.3. Construction of Plasmids for the Expression of the Heavy and Light Chains of the Chimeric Antibody against the MT-C34 Peptide

Total RNA from C24-6 mouse hybridoma cells that secrete an antibody against the MT-C34 peptide (mAb) [22] was isolated using the GeneJet RNA Purification Kit (ThermoScientific, Waltham, MA, USA). cDNA fragments encoding the variable domains of the heavy and light chains of mAbs (VH and VL, respectively) were generated according to the protocol by Meyer et al. [33]. Reverse transcription reaction was performed using MMLV Revertase (SibEnzyme, Novosibirsk, Russia), TS Oligo primer (template switch oligonucleotide), and 3′mIgH-RT primer (for the first-strand synthesis of VH cDNA) or 3′mIgK-RT primer (for the first-strand synthesis of VL cDNA). The 3′mIgH-RT primer is complementary to the highly conserved region of the heavy chain constant domain (CH) of mouse IgG, and the 3′mIgK-RT primer is complementary to the light chain kappa constant domain (CL) of mouse Ig. The nucleotide sequences of all primers used in this work are provided in Table S1. Second-strand synthesis and PCR amplification of VH and VL cDNA were performed using Pfu polymerase (SibEnzyme, Novosibirsk, Russia) with the forward primer 5′mIgG and reverse primers 3′mIgH-PCR and 3′mIgK-PCR, respectively. PCR products were separated in a 1% TAE-agarose gel and purified using the Cleanup S-Cap kit (Evrogen, Moscow, Russia), cloned into the pJET1.2 vector using the CloneJET PCR Cloning Kit (ThermoScientific, Waltham, MA, USA), and sequenced. The resulting plasmids were designated pJET1.2-H (VH) and pJET1.2-L (VL). Next, the heavy chain of chAb was constructed by combining C-24 mAb VH with the CH of Tz. For this purpose, the C-24 mAb VH fragment, together with leader peptide and Kozak sequence, was amplified on the template of the pJET1.2-H plasmid with the 5′Nhe-IgH and 3′ApaI-IgH primers and cloned into the NheI/ApaI sites of the pcDNA3.4-TzH vector [34] to generate the pMT-C34-H plasmid. The light chain of chAb was constructed by combining the C-24 mAb VL with the CL of Tz using overlap extension PCR. In the first round of PCR, VL, together with the leader peptide and Kozak sequence, was amplified on the template pJET1.2-L with the 5′NheI-IgL and 3′-IgL-34-F primers. Next, the fragment encoding the CL of Tz was amplified on the template of pcDNA3.4-TzL (unpublished, provided by Dr. T.K. Aliev, Faculty of Chemistry, Lomonosov Moscow State University) with the primers 5′-IgL-34-F and 3′-IgL-EcoRI-end. Both PCR products were purified from 1% TAE-agarose gel, mixed, and used as a template for the second round of PCR with primers 5′NheI-IgL and 3′-IgL-EcoRI-end. The resulting fragment was cloned into the NheI/EcoRI sites of the pcDNA3.4-TzL vector to generate the pMT-C34-L. The resulting plasmids pMT-C34-H and pMT-C34-L were deposited to Addgene under accession numbers 223966 and 223967, respectively.

### 2.4. Production and Purification of Chimeric Antibody

One day before transfection, 9 × 10^6^ HEK293T cells were plated into 150 mm Petri dishes in 30 mL of DMEM. The next day, cells were transfected with the expression plasmids pMT-C34-H and pMT-C34-L at a 1:2 ratio using the GenJect-39 reagent (Molecta, Moscow, Russia) according to the manufacturer’s instructions. After 5 h, the culture medium was changed to serum-free HybriS-2 medium, and the transfected cells were incubated for up to 5 days. At the end of the incubation, the culture medium was collected and centrifuged at 4 °C and 4500 rpm for 5 min.

For the purification of chAb, 120 mL of HEK293T culture supernatant from four 150 mm Petri dishes were mixed with 12 mL of 10× phosphate-buffered saline (PBS) and applied to a protein G-Sepharose column (Protein G SepFast, BioToolomics, Consett, UK), pre-equilibrated with PBS. The column was then washed with 5 volumes of PBS, and chAb was eluted with 3 volumes of 0.1 M glycine-HCl buffer (pH 2.5). Fractions were collected in tubes with 1/10 volume of 1 M Tris-HCl (pH 9) neutralization buffer. Antibody presence in each fraction was confirmed by 10% sodium dodecyl sulfate-polyacrylamide gel electrophoresis (SDS-PAGE). Fractions containing antibodies were combined and desalted using PD-10 Desalting Columns (GE Healthcare, Chicago, USA) according to the manufacturer’s instructions. The immunoglobulin concentration in each fraction was determined using a NanoPhotometer N120 (Implen, Munchen, Germany).

### 2.5. Size-Exclusion Chromatography

The Superdex 200 10/30 GL column (GE Healthcare, Chicago, IL, USA) was used for the gel filtration. The separation of samples was carried out at a rate of 0.5 mL/min in PBS on the ACTA pure 25 (1.2.0.0) system (GE Healthcare, Chicago, IL, USA). The Protein Standard Mix 44–669 kDa kit (28403842OL/AB, GE Healthcare, Chicago, IL, USA) was used for the preliminary calibration of molecular weights.

### 2.6. Polyacrylamide Gel Electrophoresis of Purified chAb

Separation of the proteins under reducing conditions was performed by 10% SDS-PAGE according to Laemmli’s protocol [35], after preheating samples in a sample loading buffer (62.5 mM Tris-HCl pH 6.8, 2% SDS, 5% 2-mercaptoethanol, 10% glycerol) at 95 °C for 5 min. In addition, proteins were resolved by 7.5% SDS-PAGE under non-reducing conditions, i.e., without 2-mercaptoethanol, or under “quasi-native” conditions when samples were loaded on the gel in 62.5 mM Tris HCl buffer pH 6.8 containing 10% glycerol but with no SDS and heat denaturation. At the end of electrophoresis, proteins in the gel were visualized by staining them with Coomassie Brilliant Blue R-250 stain (Serva, Heidelberg, Germany).

### 2.7. Western Blotting

Twenty microliters of the supernatant from transfected cells producing chAb or 0.15 μg of affinity-purified chAb were resolved by 7.5% SDS-PAGE under non-reducing conditions and then transferred onto an Immobilon-P Transfer Membrane (Merck Millipore, Burlington, MA, USA) using the Trans-Blot Turbo Transfer System (Bio-Rad, Hercules, CA, USA). The membrane was blocked with 5% skim milk in PBS containing 0.1% Tween-20 (PBS-T) for 1 h at room temperature (RT). Next, the membrane was washed three times in PBS-T and incubated with mouse anti-human IgG conjugated with horseradish peroxidase (HRP) (a gift from Prof. A.V. Filatov, NRC Institute of Immunology FMBA of Russia) at a dilution ratio of 1:5000. The membrane was washed three times with PBS-T, developed using the Immobilon Western Chemiluminescent HRP Substrate reagent (Merck Millipore, Burlington, MA, USA), and imaged on the ChemiDoc MP gel documentation system (Bio-Rad, Hercules, CA, USA).

### 2.8. Enzyme-Linked Immunosorbent Assay (ELISA)

An indirect ELISA was performed using buffers and a tetramethylbenzidine (TMB) solution purchased from CHEMA (Moscow, Russia). To sorb the MT-C34 peptide on a plastic surface, it was covalently conjugated to bovine serum albumin (BSA) by using the sulfo-succinimidyl-4-(N-maleimidomethyl) cyclohexane-1-carboxylate (sulfo-SMCC) chemical linker (ThermoScientific, Waltham, MA, USA). The resulting BSA-MT-C34 conjugate diluted in PBS at 10 μg/mL concentration was aliquoted to 100 μL per well of a 96-well ELISA-grade plate (#762071, Greiner Bio-One, Kremsmunster, Austria) overnight. The wells were blocked and washed using respective CHEMA solutions and protocols. Then 100 μL of anti-MT-C34 chAb or C24-6 mAb sampled in dilution buffer at concentrations of 1000, 500, 100, 50, 20, 10, or 5 ng/mL was added to the wells for 1 h. After washing, 100 μL of anti-human or anti-mouse (#7076, Cell Signaling, Danvers, MA, USA) IgG-HRP conjugate at a dilution of 1:15000 or 1:3000, respectively, was added to each well. The plate was incubated at RT for 1 h, and then the samples were washed and developed with 100 μL of TMB solution for 10–20 min in the dark. The reaction was stopped by adding 50 µL of 2M H_2_SO_4_. OD values were measured on an iMark Microplate reader (Bio-Rad, Hercules, CA, USA) using a 450 nm filter setting.

### 2.9. Calculation of the Equilibrium Dissociation Constant (Kd)

The method described by Friguet et al. [36] was used to calculate the *K_d_* for mAb and chAb. A fixed amount of mAb or chAb was mixed with an excess of BSA-MT-C34 antigen, which was added at various concentrations and incubated at RT for 2 h to establish dynamic equilibrium. The antibody without antigens was used as a control. Specifically, the final concentration of antibodies was 15 ng/mL (10^−10^ M), and the concentrations of the conjugated peptide (antigen) were 7.1 ng/mL, 3.55 ng/mL, 1.78 ng/mL, 0.9 ng/mL, 0.45 ng/mL, 0.23 ng/mL, or 0.12 ng/mL (10^−7^ M, 0.5 × 10^−7^ M, 2.5 × 10^−8^ M, 1.25 × 10^−8^ M, 0.63 × 10^−8^ M, 3.15 × 10^−9^ M, and 1.56 × 10^−9^ M). After incubation, the antibody–antigen mixture was transferred to a 96-well plate containing the pre-absorbed antigen and incubated for 1 h at RT. After washing, 100 μL of anti-mouse or anti-human IgG-HRP conjugates diluted 1:2000 was added to the wells for 1 h and then washed out. Next, a colorimetric reaction was carried out, and absorbance was measured at 450 nm, as described above. The *K_d_* was determined using the equation:

*A*_o_/(*A*_o_ − *A*) = *1* + *K*_d_/*a* (1)

where *A_o_* is the optical absorbance measured for an antibody in the absence of an antigen, *A* is the absorbance measured for an antibody–antigen mixture, and *a* is the molar concentration of the antigen. Linear regression graphs were created in Excel, with (*1*/*a*) × 10^–8^, *M*^–1^ values plotted on the X-axis and *A_o_*/(*A_o_* − *A*) values on the Y-axis, and a linear regression equation was obtained, in which *K_d_* equals the slope multiplied by 10^–8^ M. The measurements were performed in triplicates. The standard deviation was calculated based on the three *K_d_* values obtained for each antibody.

### 2.10. Flow Cytometry

CEM/R5 or CEM/R5/MT-C34 cells (2 × 10^5^) were washed twice with PBS and then incubated in 100 μL of PBS containing 1 μg/mL of mAb or chAb or no Ab for 1 h at 4 °C. Afterwards, the cells were washed with PBS and incubated with anti-mouse (ab97024, Abcam, Cambridge, UK) or anti-human (H10104, ThermoScientific, Waltham, MA, USA) phycoerythrin (PE)-conjugated antibody, diluted 1:250 and 1:25, respectively, for 30 min at 4 °C. The cells were washed, resuspended in 200 μL of PBS, and analyzed on a CytoFLEX S flow cytometer (Beckman Coulter, Brea, CA, USA) using a 562 nm excitation laser. The resulting data are presented using FlowJo software (X 10.0.7r2).

### 2.11. Antibody-Dependent Cell-Mediated Cytotoxicity (ADCC) Assay

CEM/R5 and CEM/R5/MT-C34 target cells were stained with 1 μM of carboxyfluorescein diacetate *N*-succinimidyl ester (CFSE, #21888, Sigma-Aldrich, St. Louis, MO, USA) for 20 min at RT, then washed twice with RPMI 1640 medium supplemented with 10% FBS. The cells were adjusted to a density of 6 × 10^5^ cells/mL in RPMI 1640/10% FBS, and 3 × 10^4^ CFSE-labeled target cells in 50 µL were plated in a U-bottom 96-well plate in triplicates. chAb was diluted to the desired concentration, and 2 µL of antibody solution was added to the cells to achieve final concentrations of 10, 1, or 0.1 μg/mL. The cells were incubated with chAb for 10 min at RT. The human cryopreserved PBMCs were thawed and used immediately in the assay as effector cells by mixing in a volume of 100 µL with target cells at a ratio of 4:1 (1.2 × 10^5^) or 7:1 (2.1 × 10^5^). In the control wells, the target and effector cells were mixed without antibodies. The plate was centrifuged for 4 min at 200× *g* and then incubated overnight (16–18 h) at 37 °C in a humidified 5% CO_2_ incubator. After incubation, the cells were washed twice with PBS and resuspended in 100 µL of PBS supplemented with 2% FBS and 1 µg/mL of propidium iodide (PI). Flow-cytometric analysis was then performed using 488 nm and 561 nm lasers to detect CFSE and PI fluorescent signals, respectively. The viability of target cells in each well was calculated as a percentage of live target cells in the overall target cell population using the following formula:

(2) $$\frac{\left[CFSE+/PI-\right]}{\left[CFSE+/PI-\right]+\left[CFSE+/PI+\right]}\times 100\%$$

where [CFSE^+^/PI^−^] is the percentage of live cells in the population of target cells and [CFSE^+^/PI^+^] is the percentage of dead cells among the target cells.

ADCC was calculated using the formula suggested by Beaudoin-Bussières et al. [37]:

(3) $$ADCC=\frac{\left[T+E\right]-\left[T+E+AB\right]}{T\;alone\;}\times 100\%$$

where [T + E] is the viability of target cells after incubation with PBMCs without an antibody, [T + E + AB] is the viability of target cells after incubation with PBMCs and an antibody, and [T alone] is the viability of target cells incubated without antibodies and PBMCs. The mean percentage ± standard deviation for each condition was calculated from triplicate wells, and each experiment was repeated at least 3 times.

### 2.12. Statistical Analysis

The unpaired *t*-test was used for comparisons of ADCC percentage between CEM/R5/MT-C34 target cells and CEM/R5 control cells for each antibody concentration. Donor’s PBMCs, the T:E ratio from three experiments, and two-tailed *p*-values were calculated using GraphPad QuickCalcs online calculator (https://www.graphpad.com/quickcalcs/ accessed on 31 July 2024). *p*-values less than 0.05 were considered to be statistically significant.

## 3. Results

### 3.1. Cloning of a Mouse Monoclonal Anti-MT-C34 Antibody cDNA and Chimerization with the Human IgG1

Previously, we have generated a C24-6 hybridoma that produces mouse monoclonal antibody recognizing HIV-1 fusion inhibitory peptides C24, C34, and MT-C34 [22]. To create a chimeric antibody capable of binding to the MT-C34 peptide and mediating human Fc-receptor function, we combined the variable regions of the C24-6 mAb with the constant regions of Tz, a humanized IgG1 antibody against Erb-b2 receptor tyrosine kinase (also known as HER-2), which is used to treat HER-2-positive tumors [38,39].

**Figure 1:**
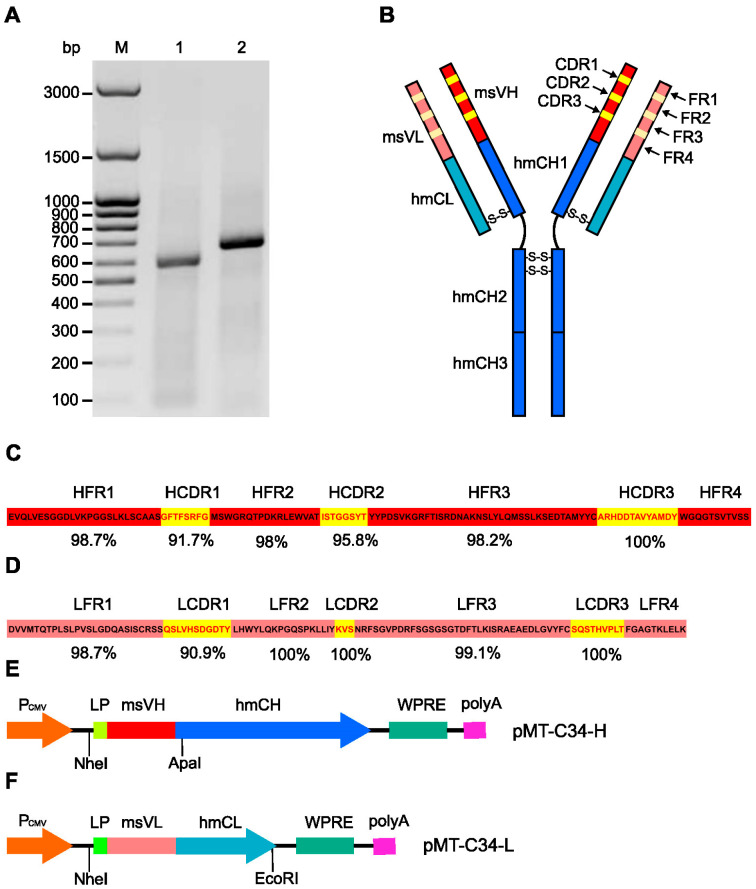
Molecular cloning of cDNA fragments encoding variable domains of a mouse monoclonal antibody against the MT-C34 peptide. (**A**) Agarose gel electrophoresis of DNA fragments amplified on a template of first strand cDNA from C24-6 hybridoma cells using light (1) and heavy (2) variable chain specific primers, (M)—DNA ladder, bp—base pair. (**B**) Domain organization of generated chimeric antibody (chAb); msVH—variable domain of mouse IgG heavy chain, msVL—variable domain of mouse Ig light chain, hmCH1, hmCH2, hmCH3—constant domains of human IgG heavy chain, hmCL—constant domain of human Ig kappa light chain, CDR—complementary determining region, FR—framework region, S-S—disulfide bond. Amino acid sequences of variable domains of C24-6 mAb heavy (**C**) and light (**D**) chains. The percentages of homology between C24-6 mAb and mouse germline V genes for each region of the variable domains of the heavy and light chains are shown below the sequence. HFR and LFR—framework regions of Ig heavy and light chain, respectively. HCDR and LCDR—complementary determining regions of Ig heavy and light chain, respectively. Schemes of the expression construct designed for the expression of chAb heavy (**E**) and light (**F**) chains. P_CMV_—cytomegalovirus early genes promoter, LP—leader peptide, msVH—variable domain of C24-6 mAb heavy chain, hmCH—constant domain of human IgG heavy chain (Tz), msVL—variable domain of anti-MT-C24 mAb kappa light chain, hmCL—constant domain of human Ig kappa light chain (Tz), WPRE—a post-transcriptional regulatory element from the woodchuck hepatitis virus, HSV TK polyA—herpes simplex virus thymidine kinase gene mRNA polyadenylation signal. NheI, ApaI, and EcoRI—restriction sites used for cloning of chimeric sequences.

For molecular cloning of the variable domains of mouse immunoglobulin chains, we used the protocol described by Meyer et al. [33]. This method relies on the usage of MMLV reverse transcriptase that can add several deoxycytidine (dC) nucleotides to the 3′ end of the transcript, generated from the reverse primer, which is complementary to the adjacent constant domain of mouse IgG. Multiple terminal dCs allow the use of a template switch forward primer with a GGG sequence at the 3′ end and amplify variable IgG domains, which may contain 5′-sequence mismatches. By following this method, we successfully RT-PCR-amplified 550 and 650 bp DNA fragments corresponding to the size of VL and VH domains of C24-6 hybridoma, respectively (Figure 1A).

The coding sequences of the amplified fragments were analyzed using the Kabat database [40] to identify the VH and VL of mAb. Then, using the IgBLAST [41] and IMGT [42] tools, the framework regions (FRs) and hypervariable complementarity-determining regions (CDR) of VH and VL mAb were identified (Figures S1 and S2). Figure 1C,D show the percentage of homology between these regions and the corresponding regions of the mouse germline V genes. The results of these analyses indicate that the obtained cDNA sequences correspond to the variable domains of the heavy and light chains of mouse IgG.

To construct a human chimeric anti-MT-C34 antibody (chAb), we replaced fragments encoding the VH and VL of Tz with the fragments encoding the VH and VL of C24-6 mAb in pcDNA3.4-TzH and pcDNA3.4-TzL plasmids, respectively. The backbone of these vectors is designed for the expression of transgenes in mammalian cells under the control of the CMV promoter. The schemes of the generated pMT-C34-H and pMT-C34-L expression plasmids and restriction sites used for cloning are schematically shown in Figure 1E,F.

### 3.2. Analysis of Purified Chimeric Antibody

**Figure 2:**
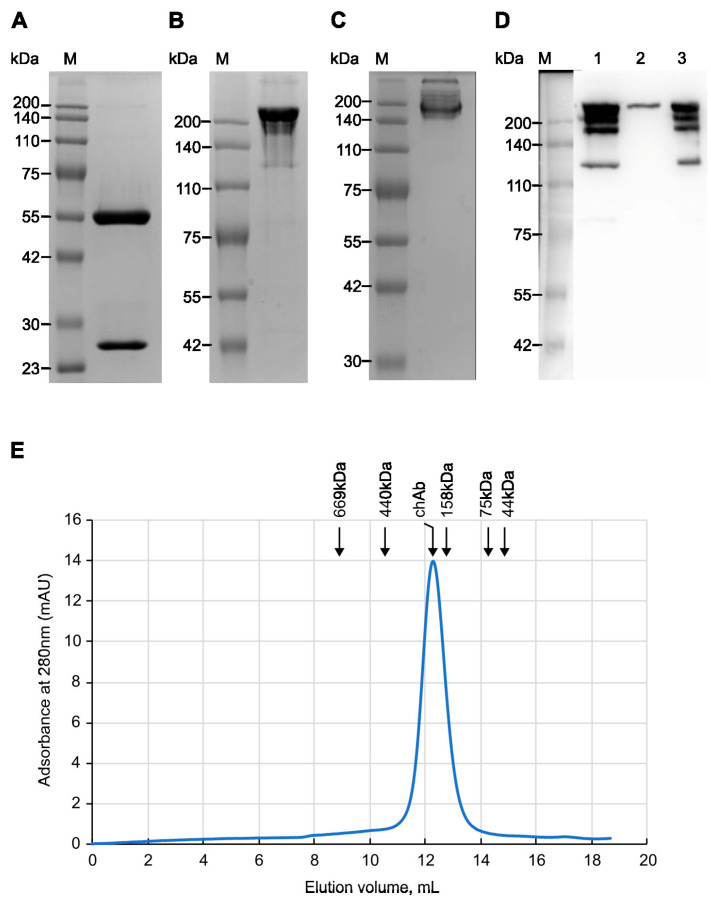
Analysis of the affinity-purified chimeric antibody (chAb). ChAb was resolved by 10% SDS-PAGE under reducing conditions (**A**), by 7.5% SDS-PAGE under non-reducing conditions (**B**), or by 7.5% SDS-PAGE under “quasi-native” conditions (**C**) followed by staining with Coomassie R-250. M—molecular weight markers (Servicebio, G2058, Wuhan, China). (**D**) Western blot analysis of affinity-purified and non-purified chAb. Lane 1: supernatant from transfected HEK293T cells secreting chAb. Lane 2: purified Ab. Lane 3: flow-through fraction. Samples were resolved by 7.5% SDS-PAGE under non-reducing conditions, blotted, and stained with an HRP-conjugated anti-human IgG antibody. M—molecular weight markers. (**E**) Analytical size-exclusion chromatography: elution profile of chAb from a Superdex 200 10/300 GL column. The elution volume of chAb—12.28 mL; the elution volumes of marker proteins: Thyroglobulin (669 kDa)—8.89 mL; Ferritin (440 kDa)—10.56 mL; Aldolase (158 kDa)—12.78 mL; Conalbumin (75 kDa)—14.23 mL; Ovalbumin (44 kDa)—14.83 mL. mAU—milli absorbance units.

To produce chAb, HEK293T cells were co-transfected with pMT-C34-H and pMT-C34-L plasmids at a 1:2 ratio. The secreted antibodies were then purified from the supernatant of transfected cells by affinity chromatography on Protein G-Sepharose. First, the purity and molecular weight of affinity-purified chAb were analyzed using SDS-PAGE. Under reducing conditions, the heavy and light chains of chAb migrated according to their expected molecular masses of 50 and 25 kDa (Figure 2A); based on band densitometry, the purity of the isolated chAb was high (>99%). Without reducing agent (Figure 2B) and SDS (Figure 2C), chAb migrated on the gel as a protein with a size of 180-200 kDa. For additional chAb verification, the supernatant from transfected HEK293T cells, purified chAb, and flowthrough fractions obtained after affinity purification were analyzed using a non-reducing SDS-PAGE followed by transferring the proteins onto a blotting membrane and probing with the anti-human IgG-HRP conjugate (Figure 2D, lanes 1, 2, and 3, respectively). The obtained data shows that chAb was efficiently purified on Protein G Sepharose (lane 2) and can be detected with anti-human secondary Ab. Running chAb under “quasi native” conditions (no SDS, no reducing agent) demonstrated that chAb migrated as an IgG monomer with a molecular weight of ~170 kDa, which is close to the estimated monomer size of 150 kDa (Figure 2C). Additionally, analytical size-exclusion chromatography of affinity-purified chAb confirmed that it was eluted from the gel-filtration column as a single peak corresponding to the IgG monomer (Figure 2E). Thus, using various protein analysis techniques, we have confirmed the purity and monomeric nature of the new anti-MT-C34 recombinant antibody.

### 3.3. Evaluating Binding Affinity of the Mouse and Chimeric Anti-MT-C34 Ab

**Figure 3:**
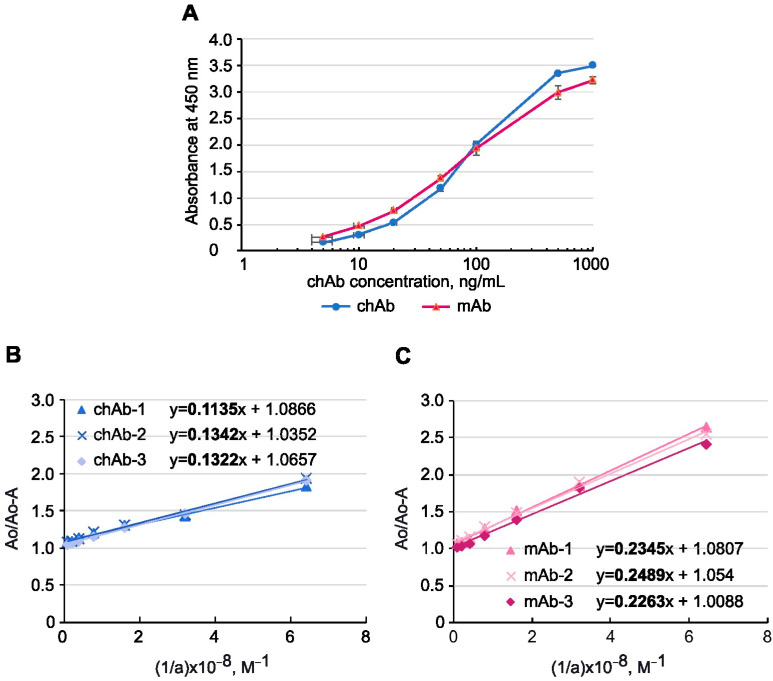
The binding affinity of anti-MT-C34 chAb and mouse mAb was measured by ELISA. (**A**) chAb and mAb titration curves obtained with MT-C34 peptide conjugated to BSA. The levels of absorbance at 450 nm were plotted against the concentration of Abs. The results are shown as mean ± standard deviation, n = 3. Determining the equilibrium dissociation constant for chAb (**B**) and mAb (**C**). A fixed amount of chAb or mAb was mixed with an excess of antigen at different concentrations, and the concentration of free antibody was measured by ELISA as in (**A**). Three linear regressions were drawn for each Ab, where Ao is the optical absorption of the antibody solution in the absence of antigen, A is the optical absorption measured in a mixture of the antibody and antigen, and a is the molar concentration of the antigen. K_d_ was determined as a slope of the linear regression multiplied by 10^−8^ M. The slope coefficients from the linear regression equations are highlighted in bold.

Substitution of mouse constant regions of IgG with those from human IgG can cause a loss or a decrease in Ab affinity. To address this issue, we compared the binding affinity of the parental mouse mAb and the generated chAb using an indirect ELISA and BSA-MT-C34 conjugate sorbed on plastic as an antigen. The obtained titration curve (Figure 3A) demonstrated that the recombinant Ab can bind to the MT-C34 peptide with an efficiency that is similar to that of mAb at nanomolar concentrations, and this binding is detected with an anti-human secondary antibody. Based on the titration curve, we estimated the chAb yield in the supernatant of transfected HEK293T cells, which was 57 mg/L on average.

Affinity is an important characteristic of a monoclonal antibody, which is measured by calculating the equilibrium dissociation constant (*K_d_*) of the antigen–antibody complex. The *K_d_* values for chAb and mAb were determined using the method described by Friguet et al. [36] and measured using an indirect ELISA after the antibody was bound to the antigen taken in excess. Figure 3B,C shows the linear regression graphs resulting from this analysis. Using the linear regression equations, the *K_d_* for the chAb–antigen complex was determined to be 1.27 × 10^−9^ ± 0.09 × 10^−9^ M, while the *K_d_* for mAb–antigen complex was 2.47 × 10^–9^ ± 0.09 × 10^–9^ M. Therefore, the replacement of mouse constant domains with human ones not only preserved chAb affinity but even increased it two-fold in comparison to the mAb, indicating that Fc-fragment substitution, which is known to influence the antigen-binding property of immunoglobulins [43,44,45], in some cases can improve it.

### 3.4. Measuring Specificity of the chAb Binding Using Flow Cytometry

**Figure 4:**
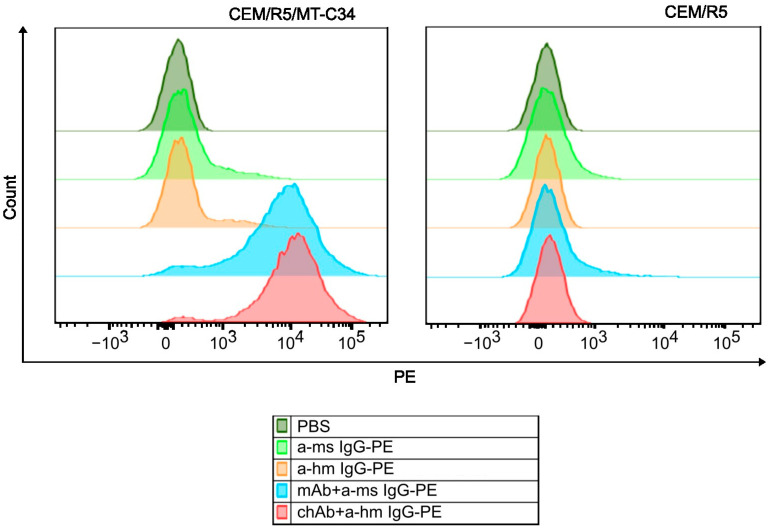
Flow cytometry analysis of chAb and mAb binding to membrane-anchored MT-C34. CEM/R5/MT-C34 (**left** panel) and CEM/R5 (**right** panel) cells were incubated with 1 µg/mL of chAb (red graph), 1 µg/mL of mAb (blue graph), or in PBS without primary antibody (orange, light green and dark green histograms), washed, and probed with phycoerythrin (PE)-labeled anti-human IgG (a-hm IgG-PE) or anti-mouse IgG (a-ms IgG-PE). After washing, cells were analyzed by flow cytometry using PE-channel.

To determine the binding specificity of the generated chAb, we compared flow cytometry staining profiles obtained for CEM/R5/MT-C34 cells and CEM parental cells. A CEM/R5/MT-C34 cell line stably expressing GPI-anchored MT-C34 peptide on the cell surface in the context of CD52 molecule was previously generated in our lab via CRISPR/Cas9-mediated knock-in into *CXCR4* locus [22]. As shown in Figure 4, both chAb and mouse mAb intensively stained CEM/R5/MT-C34 cells (Figure 4, left panel, red and blue histograms), whereas binding of these antibodies to control CEM/R5 cells was not detected (Figure 4, right panel). These data indicate that similar to the parental mouse mAb, the newly generated chAb retains a high specificity of binding to the HIV-1 peptide MT-C34, and none of the CEM surface antigens have cross-reactivity with chAb.

### 3.5. Antibody-Dependent Cellular Cytotoxic (ADCC) Function of chAb

**Figure 5:**
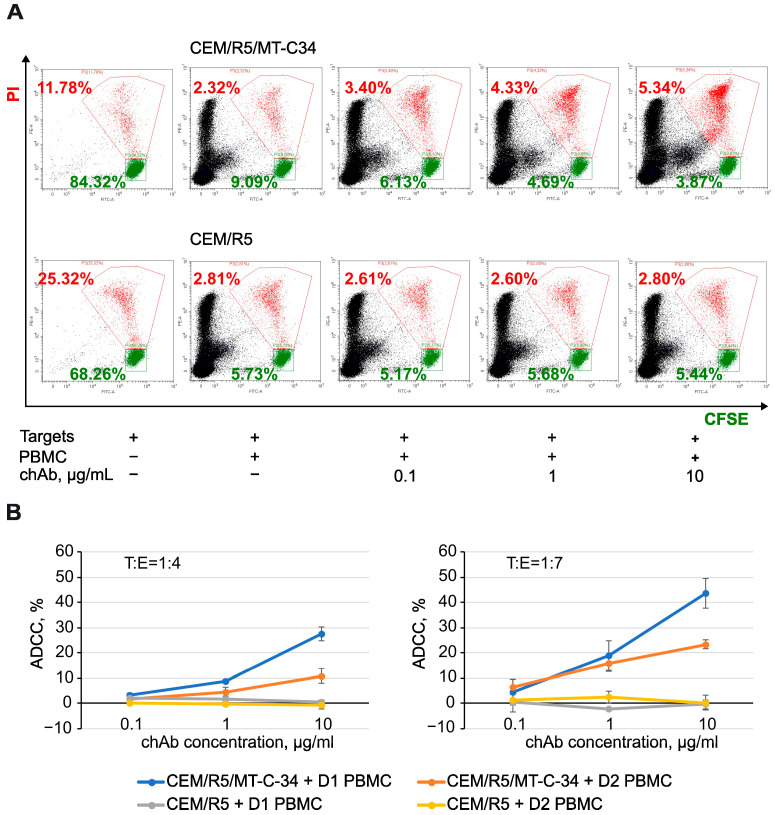
ADCC function mediated by chAb in the presence of human PBMCs. (**A**) Typical flow cytometry dot plots showing the distribution of live (green, CFSE+/PI−) CEM target cells and dead (red, CFSE+/PI+) target cells after incubation for 16 h with human PBMCs (donor 1) at a ratio of 1:7 and indicated concentrations of chAb. (**B**) Percentages of ADCC calculated from FACS data obtained with donor 1 (blue and grey lines) or donor 2 (orange and yellow lines) PBMCs. See formulas in Material and Methods for calculation. Experiments were performed at least three times in triplicate for each condition. The data are shown as the average values ± standard deviation, n = 3. Donor PBMCs displayed up to a 2-fold difference in ADCC, which may reflect variations in the level and activity of NK and other killer cells in individuals.

The fc portion of an antibody mediates its effector function, which, depending on the mechanism, is classified mainly into complement-dependent cytotoxicity (CDC), antibody-dependent cellular cytotoxicity (ADCC), and antibody-dependent cellular phagocytosis (ADCP) [29,30,31,32]. To test the function of the human portion of chAb, we selected ADCC as one of the important Ab activities elicited against infected or cancer cells. To this end, CEM/R5/MT-C34 or CEM/R5 cells covalently labeled with carboxyfluorescein diacetate N-succinimidyl ester (CFSE) were used as target cells, and PBMCs were added as effector cells at a 1:4 or 1:7 ratio with/without chAb. After overnight incubation, propidium iodide (PI) was added to the cell mixture, and samples were analyzed by flow cytometry. As demonstrated in Figure 5A, escalating doses of chAb proportionally decreased the number of live CEM/R5/MT-C34 target cells (CFSE^+^/PI^–^) and increased the number of dead CEM/R5/MT-C34 target cells (CFSE^+^/PI^+^) (top panel), however, did not alter the killing of control CEM/R5 cells (lower panel). By comparing target cell survival with and without chAb and normalizing the obtained value to a nonspecific killing effect of PBMCs on the CEM cell line, we calculated the percentage of ADCC mediated by the new recombinant chAb. Depending on the donor used, it varied from 27% to 43% when the chAb was added at a concentration of 10 µg/mL; however, no ADCC was detected against peptide-negative CEM/R5 cells (Figure 5B). The statistical analysis of ADCC data is summarized in Table 1. These data demonstrate that the chAb acquired a new property to mediate ADCC in the human effector cell system, which broadens the area of its application in the HIV field.

## 4. Discussion

Peptides derived from the heptad repeat regions of the transmembrane protein of viral Envs are potent inhibitors of viral fusion and were developed and subsequently modified/improved for the treatment of HIV-1 [14,15,20,21], SARS-CoV-2 [46,47,48], and other enveloped viruses. T20 (Enfuvirtide) and C34 (Sifuvirtide) entered HIV clinical trials in the past. The next generation of fusion inhibitory peptides, such as 2P23 [24] and MT-C34 [23] and especially their lipid modifications [17,49,50], provided a much higher level and broad protection from HIV-1 relative to the first peptides. However, the stability and solubility of peptide-lipid derivates remain problematic. As an alternative to chemical lipid anchors, we earlier developed a genetically based method for HIV inhibitory peptide GPI-anchoring and delivery to the cell surface by embedding it into the smallest human protein CD52 [51]. Using CRISPR/Cas9 knock-in of this construct into the human *CXCR4* gene, we achieved a complete resistance of T cells to both CXCR4- and CCR5-tropic HIV-1 [22].

To selectively enrich engineered HIV-1-resistant T cells, we raised mouse monoclonal and rabbit polyclonal antibodies against peptides MT-C34 and 2P23, respectively, and demonstrated that both Abs were highly specific for the peptides and efficient in sorting peptide-positive HIV-1 resistant CEM T cells and primary lymphocytes. C24-6 hybridoma recognized three related peptides C24, C34, and MT-C34 [22]. C24-6 mAb, however, did not directly affect HIV-1 transmission or interfere with the protective potency of GPI-anchored MT-C34 (unpublished data), which can be explained by the inaccessibility of HR1 regions of gp41 for the mAb and by the ability of the MT-C34–mAb complex to bind the HR2 region of gp41 and inhibit fusion. Thus, the generated C24-6 mAb was applicable only for immunofluorescent staining and live cell sorting.

In this study, based on hybridoma C24-6, we created a recombinant chAb by fusing antigen-specific regions of mouse mAb with the constant regions of the humanized IgG1 Tz. The resulting antibody retained high specificity towards the MT-C34 peptide and displayed a two-fold higher affinity than the mouse counterpart. Unlike mouse mAb, chAb mediated potent ADCC with the human PBMCs used as killer cells. Together with the ease of production, no need to maintain hybridoma cells, antibody functionality, and low immunogenicity in the human system, the generated recombinant chAb offers a more convenient affinity reagent with a broader range of applications that we discuss below.

Advances in the development of programmable nucleases have led to attempts to eradicate HIV or achieve a functional cure by knocking out viral entry coreceptor CCR5 using ZNF [52,53] or CRISPR/Cas9 nuclease [54] or by expressing HIV-1 fusion inhibitory peptides, delivered to target T cells via lentiviral transduction [19,20,21] or CRISPR/Cas9-mediated knock-in technology [22]. The ultimate goal of all these studies was to generate as many as possible HIV-1 resistant T cells ex vivo and return them to the patients. In practice, treated lymphocytes are reinfused into individuals without selection, which, of course, reduces the efficacy of gene therapy. In this context, chAb is a valuable reagent that, in combination with the MT-C34 peptide delivery tool, could be used for a specific selection of HIV-1-resistant T cells. A high-affinity nature of antibodies suggests that they will enter a human organism in conjunction with selected cells and, upon repeating procedures often required to achieve a therapeutic effect, could induce an anti-mouse antibody response [55]. In this regard, chAb will be a less immunogenic reagent than the mouse antibody. The next procedure of chAb humanization, i.e., the substitution of mouse FRs with human analogs, will make it the least immunogenic and more suitable for clinical application.

Another attractive application for chAb is its combination with CAR cell therapeutics, which can pursue two goals. First, the widely used practice of bulk PBMC transduction with CAR constructs designed to treat HIV infection makes killer cells (CD4^+^) vulnerable to HIV-1 infection, which will certainly reduce the therapeutic effect. Introduction of a short cassette for the expression of MT-C34 in the context of the CD52 protein [22] into a CAR construct together with the chAb immunoreagent can make the CAR T cell population resistant to HIV-1 and enrich it using FACS-sorting before infusion to a recipient. This strategy can be applied not only for anti-HIV CARs but also to treat malignancies in HIV patients. Second, multiple clinical reports demonstrated that CAR cells can be transformed into leukemic cells [56] due to, for instance, retro/lentiviral delivery of CAR into the 3′UTR of the PBX2 gene [57] or piggyBac transposon usage [58]. This raises safety concerns about CAR therapy and the development of an additional layer of CAR cell elimination that is not triggered by the allogenic immune rejection mechanism. In this respect, chAb, which demonstrated a good level of ADCC with human PBMCs, can serve as a specific tool to trace and subsequently eliminate MT-C34 engineered CAR cells, which, after accomplishing their job, no longer needed to be present in a living organism.

In the current study, however, we neither evaluated the possibility of MT-C34 chAb application in vivo nor fully characterized its effector function. Therefore, further research is needed to determine the Fc-mediated activity of chAb, such as CDC and ADCP, which can also contribute to the elimination of engineered cells in vivo. Our long-term future direction is to combine CRISPR/Cas-based T cell resistance generation with an anti-HIV-1 CAR cell therapy to potentiate the elimination of latent virus reservoirs, and the new anti-MT-C34 chAb will be a key element providing efficacy and safety of HIV therapy.

## 5. Conclusions

In summary, we generated chAb against HIV-1 fusion inhibitory peptide MT-C34, which is highly specific, easily produced and purified, and has a functional Fc-fragment that mediates ADCC. Genetic constructs for the production of chAb are accessible through Addgene, and we believe that this chAb would be a good asset in the field of HIV research/treatment and CAR-based therapies.

## Figures and Tables

**Table 1:** Statistical analysis of ADCC mediated by chAb. *p*-values were calculated for CEM/R5/MT-C34 + D1 PBMCs vs CEM/R5 + D1 PBMCs and CEM/R5/MT-C-34 + D2 PBMCs vs CEM/R5 + D2 PBMCs experimental points. *p*-values are marked with an asterisk (*) when the difference in ADCC is statistically significant.

T:E = 1:4						
chAb Concentration, µg/mL	0.1		1		10	
	ADCC, %	*p*-value	ADCC, %	*p*-value	ADCC, %	*p*-value
CEM/R5/MT-C34 + D1 PBMCs	3.28 ± 0.32	0.2285	8.88 ± 0.9	0.001 *	27.88 ± 2.76	<0.0001 *
CEM/R5/MT-C-34 + D2 PBMCs	1.79 ± 2.11	0.2423	4.3 ± 2.04	0.027 *	10.9 ± 3.00	0.0047 *
CEM/R5 + D1 PBMCs	1.99 ± 1.54	-	1.91 ± 1.08	-	0.71 ± 0.41	-
CEM/R5 + D2 PBMCs	0.11 ± 0.23	-	−0.06 ± 0.86	-	−0.4 ± 1.68	-
**T:E = 1:7**						
**chAb concentration, µg/mL**	**0.1**		**1**		**10**	
	ADCC, %	*p*-value	ADCC, %	*p*-value	ADCC, %	*p*-value
CEM/R5/MT-C34 + D1 PBMC	4.39 ± 5.14	0.2607	18.83 ± 5.88	0.0034 *	43.62 ± 5.93	0.0003 *
CEM/R5/MT-C-34 + D2 PBMC	6.57 ± 2.93	0.1795	15.85 ± 3.19	0.004 *	23.27 ± 1.78	0.0003 *
CEM/R5 + D1 PBMC	0.49 ± 0.46	-	−2.29 ± 0.15	-	−0.28 ± 1.77	-
CEM/R5 + D2 PBMC	1.39 ± 4.68	-	2.30 ± 2.34	-	0.22 ± 2.99	-

## Data Availability

Data is contained within the article or supplementary material. The dataset is available on request from the authors. Plasmids pMT-C34-M-H and pMT-C34-L were deposited to Addgene under accession numbers 223966 and 223967.

## Supplementary Materials

The following supporting information can be downloaded at: https://www.mdpi.com/article/10.3390/biology13090675/s1, Figure S1: Result of IgBLAST analysis of C24-6 mAb heavy chain variable domain; Figure S2. Result of IgBLAST analysis of C24-6 mAb light chain variable domain; Table S1: Primers used to construct pMT-C34-H and pMT-C34-L.
