# Supplementary material for: A New Chimeric Antibody against the HIV-1 Fusion Inhibitory Peptide MT-C34 with a High Affinity and Fc-Mediated Cellular Cytotoxicity
Source: Biology (Basel). 2024 Aug 29;13(9):675. doi: 10.3390/biology13090675 (PMC11428423; doi:10.3390/biology13090675)
Supplement: Supplementary file 1 [file biology-13-00675-s001.zip › biology-3163530-supplementary.pdf]

Table S1. Primers used to construct pMT-C34-H and pMT-C34-L.

Name	Sequence; 5′-> 3′	Ref.
TS Oligo	AAGCAGTGGTATCAACGCAGAGTACATGrGrGr	[33]
3'mIgK-RT	TTGTCGTTCACTGCCATCAATC	[33]
3'mIgH-RT	AGCTGGGAAGGTGTGCACAC	[33]
5'mIgG	AAGCAGTGGTATCAACGCAGAG	[33]
3'mIgK-PCR	ACATTGATGTCTTTGGGGTAGAAG	[33]
3'mIgH-PCR	GGGATCCAGAGTTCCAGGTC	[33]
5′ NheI-IgH	GGGCTAGCCACCATGAACTTCGGGC	
3′ApaI-IgH	GATGGGCCCTTCGTGCTGGCTGAGGAGACGGTGACTGAGG	
5′NheI-IgL	TAGCTAGCCACCATGAAGTTGCCTGTTAGGCT	
3′IgL-34-F	CCACAGTCCGTTTCAGCTCCAGCTTGGTCCCAG	
5′IgL-34-F	GGAGCTGAAACGGACTGTGGCTGCACCA	
3′-IgL-EcoRI-end	GACGGAGCTCGAATTCGGATCC	

Query= C24-6 VH

Length=360

Sequences producing significant alignments:

	Score (Bits)	E Value
IGHV5-6*01germline gene	441	1e-125
IGHV5-6-1*01germline gene	441	1e-125
IGHV5-6*02germline gene	433	2e-123
IGHJ4*01germline gene	93.0	3e-23
IGHJ2*02germline gene	46.8	2e-09
IGHJ2*01germline gene	41.1	1e-07

Domain classification requested: imgt

V-(D)-J rearrangement summary for query sequence (multiple equivalent top matches, if present, are separated by a comma):

Top V gene match	Top D gene match	Top J gene match	Chain type	stop codon	V-J frame	Productive	Strand	V frame shift
IGHV5-6*01,IGHV5-6-1*01	N/A	IGHJ4*01	VH	No	In-frame	Yes	+	No

V-(D)-J junction details based on top germline gene matches:

V region end	V-D junction*	D region	D-J junction*	J region start
AGACA			TGACGATACTGCGGTT	TATGC

Sub-region sequence details:

	Nucleotide sequence	Translation	Start	End
CDR3	GCAAGACATGACGATACTGCGGTTTATGCTATGGACTAC	ARHDDTAVYAMDY	289	327

Alignment summary between query and top germline V gene hit:

	from	to	length	matches	mismatches	gaps	identity(%)
FR1-IMGT	1	75	75	74	1	0	98.7
CDR1-IMGT	76	99	24	22	2	0	91.7
FR2-IMGT	100	150	51	50	1	0	98
CDR2-IMGT	151	174	24	23	1	0	95.8
FR3-IMGT	175	288	114	112	2	0	98.2
CDR3-IMGT (germline)	289	296	8	8	0	0	100
Total			296	289	7	0	97.6

Alignments

```
                                       <--------------------------------FR1-IMGT---------------------------------><-------CDR1-IM
                                        E  V  Q  L  V  E  S  G  G  D  L  V  K  P  G  G  S  L  K  L  S  C  A  A  S  G  F  T  F  S
                    Query_1       1    GAGGTGCAGTTGGTGGAGTCTGGGGGAGACTTAGTGAAGCCTGGAGGGTCCCTGAAACTCTCCTGTGCAGCCTCTGGATTCACTTTCAGT  90
V  97.6% (289/296)  IGHV5-6*01    1    .........C................................................................................  90
                                        E  V  Q  L  V  E  S  G  G  D  L  V  K  P  G  G  S  L  K  L  S  C  A  A  S  G  F  T  F  S
V  97.6% (289/296)  IGHV5-6-1*01  1    .........C................................................................................  90
V  96.9% (286/295)  IGHV5-6*02    1    ..A...A..C................................................................................  90

                                       GT------><--------------------FR2-IMGT---------------------><-------CDR2-IMGT------><-----
                                        R  F  G  M  S  W  G  R  Q  T  P  D  K  R  L  E  W  V  A  T  I  S  T  G  G  S  Y  T  Y  Y
                    Query_1       91   AGGTTTGGCATGTCTTGGGGTCGCCAGACTCCAGACAAGAGGCTGGAGTGGGTCGCAACCATTAGTACTGGTGGTAGTTACACCTACTAT  180
V  97.6% (289/296)  IGHV5-6*01    91   ..C.A..............T...............................................G......................  180
                                        S  Y  G  M  S  W  V  R  Q  T  P  D  K  R  L  E  W  V  A  T  I  S  S  G  G  S  Y  T  Y  Y
V  97.6% (289/296)  IGHV5-6-1*01  91   ..C.A..............T...............................................G......................  180
V  96.9% (286/295)  IGHV5-6*02    91   ..C.A..............T...............................................G......................  180

                                       ----------------------------------------------FR3-IMGT------------------------------------
                                        P  D  S  V  K  G  R  F  T  I  S  R  D  N  A  K  N  S  L  Y  L  Q  M  S  S  L  K  S  E  D
                    Query_1       181  CCAGACAGTGTGAAGGGGCGATTCACCATCTCCAGAGACAATGCCAAGAATTCCCTGTACCTGCAAATGAGCAGTCTGAAGTCTGAGGAC  270
V  97.6% (289/296)  IGHV5-6*01    181  ..................................................CA......................................  270
                                        P  D  S  V  K  G  R  F  T  I  S  R  D  N  A  K  N  T  L  Y  L  Q  M  S  S  L  K  S  E  D
V  97.6% (289/296)  IGHV5-6-1*01  181  ..................................................CA......................................  270
V  96.9% (286/295)  IGHV5-6*02    181  ..................................................CA......................................  270

                                       -----------------><--------------CDR3-IMGT--------------><-----------FR4-IMGT------------>
                                        T  A  M  Y  Y  C  A  R  H  D  D  T  A  V  Y  A  M  D  Y  W  G  Q  G  T  S  V  T  V  S  S
                    Query_1       271  ACAGCCATGTATTACTGTGCAAGACATGACGATACTGCGGTTTATGCTATGGACTACTGGGGTCAAGGAACCTCAGTCACCGTCTCCTCA  360
V  97.6% (289/296)  IGHV5-6*01    271  ..........................----------------------------------------------------------------  296
                                        T  A  M  Y  Y  C  A  R
V  97.6% (289/296)  IGHV5-6-1*01  271  ..........................----------------------------------------------------------------  296
V  96.9% (286/295)  IGHV5-6*02    271  .........................-----------------------------------------------------------------  295
J  100.0% (48/48)   IGHJ4*01      6    ------------------------------------------................................................  53
J  87.2% (34/39)    IGHJ2*02      9    ---------------------------------------------------...........C.....C.....TC....A.........  47
J  84.6% (33/39)    IGHJ2*01      9    ---------------------------------------------------...........C.....C...A.TC....A.........  47
```

Figure S1. Result of IgBLAST analysis of C24-6 mAb heavy chain variable domain.

Query= C24-6 VL

Length=336

Sequences producing significant alignments:	Score (Bits)	E Value
IGKV1-110*01germline gene	452	5e-129
IGKV1-110*02germline gene	430	2e-122
IGKV1-117*01germline gene	427	2e-121
IGKJ5*01germline gene	71.8	6e-17
IGKJ2*02germline gene	35.3	6e-06
IGKJ2*01germline gene	35.3	6e-06

Domain classification requested: imgt

V-(D)-J rearrangement summary for query sequence (multiple equivalent top matches, if present, are separated by a comma):

Top V gene match	Top J gene match	Chain type	stop codon	V-J frame	Productive	Strand	V frame shift
IGKV1-110*01	IGKJ5*01	VK	No	In-frame	Yes	+	No

V-(D)-J junction details based on top germline gene matches:

V region end	V-J junction*	J region start
GTTCC		GCTCA

Sub-region sequence details:

	Nucleotide sequence	Translation	Start	End
CDR3	TCTCAAAGTACACATGTTCCGCTCACG	SQSTHVPLT	280	306

Alignment summary between query and top germline V gene hit:

	from	to	length	matches	mismatches	gaps	identity(%)
FR1-IMGT	1	78	78	77	1	0	98.7
CDR1-IMGT	79	111	33	30	3	0	90.9
FR2-IMGT	112	162	51	51	0	0	100
CDR2-IMGT	163	171	9	9	0	0	100
FR3-IMGT	172	279	108	107	1	0	99.1
CDR3-IMGT (germline)	280	299	20	20	0	0	100
Total			299	294	5	0	98.3

Alignments

```
                                       <---------------------------------FR1-IMGT----------------------------------><-----------
                                        D  V  V  M  T  Q  T  P  L  S  L  P  V  S  L  G  D  Q  A  S  I  S  C  R  S  S  Q  S  L  V
                    Query_1       1    GATGTTGTGATGACCCAAACTCCACTCTCCCTGCCTGTCAGTCTTGGAGATCAAGCCTCCATCTCTTGTAGATCTAGTCAGAGCCTTGTA  90
V  98.3% (294/299)  IGKV1-110*01  1    ....................................................................C.....................  90
                                        D  V  V  M  T  Q  T  P  L  S  L  P  V  S  L  G  D  Q  A  S  I  S  C  R  S  S  Q  S  L  V
V  96.0% (287/299)  IGKV1-110*02  1    ........................................................T...........C.....................  90
V  95.7% (286/299)  IGKV1-117*01  1    ......T.............................................................C...............A.....  90

                                       CDR1-IMGT----------->--------------------FR2-IMGT---------------------><CDR2-IM><--------
                                        H  S  D  G  D  T  Y  L  H  W  Y  L  Q  K  P  G  Q  S  P  K  L  L  I  Y  K  V  S  N  R  F
                    Query_1       91   CATAGTGATGGAGACACCTATTTACATTGGTACCTGCAGAAGCCAGGCCAGTCTCCAAAGCTCCTGATCTACAAAGTTTCCAACCGATTT  180
V  98.3% (294/299)  IGKV1-110*01  91   ..C...A.....A.............................................................................  180
                                        H  S  N  G  N  T  Y  L  H  W  Y  L  Q  K  P  G  Q  S  P  K  L  L  I  Y  K  V  S  N  R  F
V  96.0% (287/299)  IGKV1-110*02  91   ..C..CA.....A...........T................................................GG...............  180
V  95.7% (286/299)  IGKV1-117*01  91   ......A.....A...........G.A..............A................................................  180

                                       ----------------------------------------FR3-IMGT------------------------------------------
                                        S  G  V  P  D  R  F  S  G  S  G  S  G  T  D  F  T  L  K  I  S  R  A  E  A  E  D  L  G  V
                    Query_1       181  TCTGGGGTCCCAGACAGGTTCAGTGGCAGTGGATCAGGGACAGATTTCACACTCAAGATCAGCAGAGCGGAGGCTGAGGATCTGGGAGTT  270
V  98.3% (294/299)  IGKV1-110*01  181  ...................................................................T......................  270
                                        S  G  V  P  D  R  F  S  G  S  G  S  G  T  D  F  T  L  K  I  S  R  V  E  A  E  D  L  G  V
V  96.0% (287/299)  IGKV1-110*02  181  ...................................................................T......................  270
V  95.7% (286/299)  IGKV1-117*01  181  ...................................................................T......................  270

                                       --------><--------CDR3-IMGT--------><----------FR4-IMGT---------->
                                        Y  F  C  S  Q  S  T  H  V  P  L  T  F  G  A  G  T  K  L  E  L  K
                    Query_1       271  TATTTCTGCTCTCAAAGTACACATGTTCCGCTCACGTTCGGTGCTGGGACCAAGCTGGAGCTGAAA  336
V  98.3% (294/299)  IGKV1-110*01  271  .............................-------------------------------------  299
                                        Y  F  C  S  Q  S  T  H  V  P
V  96.0% (287/299)  IGKV1-110*02  271  ..........T....G.............-------------------------------------  299
V  95.7% (286/299)  IGKV1-117*01  271  ....A.....T....G..T..........-------------------------------------  299
J  100.0% (37/37)   IGKJ5*01      1    -----------------------------.....................................  37
J  84.8% (28/33)    IGKJ2*02      6    ---------------------------------........AT.G..............AA.....  38
J  88.9% (24/27)    IGKJ2*01      5    --------------------------------.........A.GG..............-------  31
```

Figure S2. Result of IgBLAST analysis of C24-6 mAb light chain variable domain.
